# Scientia Pharmaceutica, Autorenhinweise 2015

**DOI:** 10.3797/scipharm.aut-15-01

**Published:** 2014-11-22

Die Zeitschrift **Scientia Pharmaceutica** (www.scipharm.at) erscheint vierteljährlich jeweils am Quartalsende und ist ein Medium zur Publikation von Originalarbeiten, Kurzmitteilungen und ausgewählten Übersichtsarbeiten aus allen wissenschaftlichen Disziplinen der Pharmazie und angrenzenden Gebieten sowie der pharmazeutischen Praxis.

Scientia Pharmaceutica (ISSN 0036-8709, e-ISSN 2218-0532) wird in zahlreichen Datenbanken laufend indexiert; darunter auch: Chemical Abstracts (SciFinder), Scopus, PubMed, PubMed Central, Asian Science Citation Index, International Pharmaceutical Abstracts, Google Scholar, Natural Product Updates, CAB Abstracts, EBSCO, Chimica, ChemInform, ProQuest, Embase / Excerpta Medica, Medicinal and Aromatic Plants Abstracts, etc.

Manuskripte können entweder in Deutsch oder Englisch verfasst werden und sollten möglichst elektronisch per E-Mail an office@scipharm.at eingereicht werden. Der Manuskripteingang wird dem Korrespondenzautor per E-Mail bestätigt.

Die Schriftleitung behält sich vor, Arbeiten mit groben sprachlichen, formalen oder inhaltlichen Mängeln abzulehnen (siehe auch Kriterien für eine sofortige Ablehnung). Vor der Einreichung von Übersichtsarbeiten ist eine Zusammenfassung der Reaktion zu schicken. Es werden nur unveröffentlichte Arbeiten angenommen; sind Teilergebnisse bereits veröffentlicht, so sind diese korrekt zu zitieren und die entsprechenden Arbeiten beim Einreichen mitzuschicken.

Die eingelangten Manuskripte werden einem Begutachtungsverfahren (peer review) unterzogen, das ein zugeteilter Fachredakteur mit Hilfe von externen Gutachtern leitet. Bei der Einreichung sollten 3–5 unabhängige und kompetente Gutachter vorgeschlagen werden (komplette Anschrift und E-Mail-Adressen sind erforderlich). Die endgültige Entscheidung obliegt der Schriftleitung.

Mit der Einreichung eines Manuskriptes bestätigt der Autor, die Erlaubnis seiner Co-Autoren zu besitzen, folgende Abmachungen zu tätigen. Er bestätigt, in seinem Namen so wie im Namen der Co-Autoren, dass…

die eingereichte Arbeit weder in einer anderen Zeitschrift veröffentlicht wurde noch sich unter Begutachtung befindet, noch gegen Urheber- oder andere Rechte dritter verstößt;

er der einzige Autor ist / sie die einzigen Autoren sind und die Befugnis besitzt / besitzen, diese Abmachung einzugehen und Lizenzrechte der Österreichischen Apothekerverlagsges. m. b. H. zu gewähren;

die Arbeit weder rechtwidrig noch verleumderisch ist und die Veröffentlichung gegen keinerlei Verträge, Verschwiegenheitsabkommen oder Geheimsachen verstößt;

er / sie für die Vollständigkeit und Rechtschaffenheit des Artikels gesorgt hat / haben. Nach bestem Wissen und Gewissen des Autors / der Autoren so wie nach dem Stand der Wissenschaft sind die in dem Artikel getätigten Aussagen wahr und die genaue und sorgfältige Wiederholung der angegebenen Arbeitsvorschriften führt weder zu Verletzungen noch zu Krankheit;

die eingereichte Arbeit – sofern angenommen – unter den Creative Commons 3.0-Lizenzbestimmungen (http://creativecommons.org/licenses/by/3.0/) veröffentlicht wird und der Österreichischen Apothekerverlagsges. m. b. H. lizenziert wird. Zusammengefasst bedeutet dies, dass der Autor / die Autoren das Copyright behalten und die Arbeit open access veröffentlicht wird: Es ist jedermann gestattet, das Werk zu vervielfältigen, zu verbreiten und öffentlich zugänglich zu machen; Abwandlungen bzw. Bearbeitungen des Inhaltes anzufertigen; kommerziell zu nutzen zu den folgenden Bedingungen: Sie müssen den Namen des Autors/Rechteinhabers in der von ihm festgelegten Weise nennen (zitieren) und im Falle einer Verbreitung müssen Sie anderen die Lizenzbedingungen, unter welche dieses Werk fällt, mitteilen. Jede der vorgenannten Bedingungen kann aufgehoben werden, sofern dies der Rechteinhaber einwilligt.

Es gibt keinerlei Gebühren oder Kosten, die dem Autor bzw. seiner Institution im Rahmen der Veröffentlichung eines Artikels anfallen. Die Onlineversion der Zeitschrift kann kostenlos gelesen werden; Informationen zum Abonnement der Druckversion sind online verfügbar: www.scipharm.at/bestellung

## Checkliste für die Einreichung von Manuskripten

□ Wurde die aktuelle Version der Autorenrichtlinien von www.scipharm.at/autoren heruntergeladen?
□ Wurde das Manuskript entsprechend den Autorenrichtlinien, möglichst mit Hilfe der MS Word-Manuskriptvorlage erstellt?
□ Überprüfung auf Rechtschreibfehler? (Externe) Sprachkorrekturen?
□ Einzelne MS Word-Datei eingereicht? Alle Tabellen, Grafiken und Schemata im Hochformat in geeigneter Qualität (hohe Auflösung) und an der entsprechenden Stelle im Manuskript eingefügt?
□ E-Mail-Adresse der Autoren eingefügt (zumindest Korrespondenzautor)?
□ Vollständige Namen aller Autoren (kompletter Vor- und Nachname)?
□ Kriterien für sofortige Ablehnungen gelesen?
□ Korrekte Formatierung der Zitate?
□ Begleitschreiben mit allen relevanten Informationen für die Schriftleitung (ethische Stellungnahmen; unabhängige Gutachtervorschläge inkl. E-Mail- und Postadresse; etc.)

## Hinweise zur Manuskriptgestaltung

Es wird dringend empfohlen vor Erstellung des Manuskriptes, aktuelle Artikel der Zeitschrift als Referenz für die Formatierung heranzuziehen. Außerdem steht eine MS Word-Vorlage zur Verfügung, die unter http://www.scipharm.at/autoren heruntergeladen werden kann.

### Abbildungen und Tabellen

Abbildungen, Schemata, Grafiken und Tabellen müssen in den Text in geeigneter Größe und Qualität ausschließlich im Hochformat eingebettet werden. Bitte beachten Sie, dass die Lesbarkeit auch noch nach der produktionsbedingten Größenreduktion auf 70% gegeben sein muss.

Chemische Strukturen müssen mit einem geeigneten Programm (vorzugsweise ACD/ChemSketch) gefertigt sein und eine einheitliche Größe und Form innerhalb des Manuskripts aufweisen.

### Nomenklatur

Die chemische Nomenklatur muss den IUPAC Regeln folgen und sollte mit einem professionellen Computerprogramm kontrolliert werden; Die botanische Nomenklatur muss in Übereinstimmung mit dem “International Code” oder “Englers Syllabus der Pflanzennamen” erfolgen.

### Charakterisierung neuer Substanzen

Alle neu erhaltenen Verbindungen bedürfen einer sorgfältigen und dem stand der Technik entsprechenden Analyse; typischerweise: ^1^H-NMR, ^13^C-NMR, MS (inkl. Fragmentierungsmuster bei EI-MS), Elementaranalyse; Schmelzpunkt für feste Substanzen. Weitere Techniken wie z. B. IR, HRMS, X-NMR, 2D NMR, Röntgenstrukturanalyse, HPLC, etc. können erforderlich sein und sollten nach Möglichkeit angewandt werden. Originalspektren sind – sofern nicht bereits als Zusatzmaterial eingereicht – auf Verlangen nachzureichen.

Für pharmakologische Untersuchungen von Pflanzenextrakten sind charakteristische DC/HPLC-Chromatogramme erforderlich (fingerprint) um z. B. die Vergleichbarkeit der Ergebnisse zu gewähren.

#### Fiktives Beispiel:

Farblose Kristalle FP 118–120 °C (wässr. Ethanol). MS (*m/z*, %): 202 (M^+^, 3), 184 (36), 73 (100). IR (KBr), ν, cm^−1^: 1683, 1645. ^1^H-NMR (500 MHz, CDCl_3_, TMS): δ 8.74–8.80 (m, 2H, H-3,5), 8.18 (dd, ^3^*J*_H2,H1_ = 7.7 Hz, ^4^*J*_H2,H7_ = 1.1 Hz, 1H, H2), 7.68 (s, 1H, H-6), … ^13^C-NMR (125 MHz, DMSO-*d*_6_, TMS): δ 175.4 (COOH), 136.3 (C-7), 134.1 (C_q_), 128.0 (CH), 127.6 (CH), 54.3 (CH_2_), … HRMS: Berechnet für C_11_H_10_N_2_O_2_: 202.0742. Gefunden: 202.0746. Analyse Berechnet für C_11_H_10_N_2_O_2_: C, 65.34; H, 4.98; N, 13.85. Gefunden: C, 65.04; H, 5.22; N, 13.60.

### Sprachkorrekturen

Die Verantwortung für sprachlich einwandfreie Arbeiten liegt bei den Autoren. Bei Veröffentlichungen in einer anderen als der Muttersprache sind möglichst externe Institutionen zu beauftragen und die entsprechenden Referenzen im Begleitschreiben bei der Einreichung mitzuteilen. Diverse Sprachinstitute sind unter www.scipharm.at/autoren verklinkt. Sci Pharm spricht jedoch ausdrücklich keine Empfehlung für eine bestimmte Firma aus und hat keinerlei finanzielle Verbindungen diesbezüglich.

### Zusatzmaterial

Spektren, umfangreiche Tabellen und Abbildungen können bzw. sollen als Zusatzmaterial in der elektronischen Ausgabe veröffentlicht werden.

### Ethische Stellungnahmen der Autoren

#### Interessenskonflikte

Ein Interessenskonflikt besteht sobald ein Individuum (oder dessen Institution) eine finanzielle, persönliche oder berufsmäßige Beziehung zu anderen Personen oder Institutionen besitzt, die seine Entscheidungen und Handlungen beeinflussen könnte. Interessenskonflikte entstehen aus solchen Situationen unabhängig davon, ob die Entscheidung konkret oder möglicherweise beeinflusst ist.

#### Beispiele für eine Stellungnahme

Die Autoren bestätigen ihre Unabhängigkeit. / ABC besitzt ein Patent für … / DEF erhält als Berater ein Honorar von der Firma XYZ …

### Patientenaufklärung und –einverständnis, Befürwortung Ethikkommission

Patienten haben ein Recht auf Privatsphäre, gegen das nicht verstoßen werden darf. Jegliche Information, die den Patienten identifizieren könnte (z. B. Namen, Initialen, Krankenhausnummern, etc.) dürfen nicht veröffentlicht werden, sofern dies nicht aus wissenschaftlicher Sicht erforderlich ist und der betreffende Patient (bzw. seine Eltern oder sein Vormund) seine schriftliche Zustimmung gibt.

Bei Untersuchungen von Menschen muss im Abschnitt „Experimentelles” bestätigt werden, dass die ethischen Standards der Helsinki-Deklaration von 1964 (erneuert 2000, http://www.wma.net/e/policy/b3.htm) eingehalten wurden und dass eine Zustimmung des zuständigen Ethikkomitees vorliegt.

#### Tierrechte

Bei Tierexperimenten muss im Abschnitt „Experimentelles” bestätigt werden, dass die nationalen und internationalen ethischen Standards eingehalten wurden und dass eine Zustimmung des zuständigen Ethikkomitees vorliegt.

### Literatur

Literaturverweise im Text werden fortlaufend in eckigen Klammern nummeriert z. B. [2–4] und im Abschnitt “Literatur” aufgelistet. Schriftformat ist Arial 10, normal, einzeilig. Fuß- bzw. Endnoten dürfen nicht verwendet werden. Die Zitierung der Literatur erfolgt in Anlehnung an den “Vancouver style” [1] wie unten dargestellt (Bücher [2], Zeitschriften [3, 4]).

□ *Autor*: In der ersten Zeile eines Zeitschriftenzitates sind alle Autoren in der Originalreihenfolge anzugeben. Jedem Nachnamen werden die Initialen seiner Vor- bzw. Mittelnamen (max. 2) nachgestellt. Einzelne Autoren werden mittels Beistrich getrennt. Nach dem letzten Autor erfolgt ein Punkt und ein weicher Zeilenumbruch (durch gleichzeitiges Drücken der Enter- und Shifttaste)
□ *Titel*: Der Titel wird wie im Original wiedergegeben, gefolgt von einem Punkt und einem weichen Zeilenumbruch.
□ *Zeitschrift*: Der Titel wird abgekürzt; alle Wörter beginnen mit einem Großbuchstaben; keine Punkte außer nach dem letzten Wort gefolgt von einem Leerzeichen. Danach das Erscheinungsjahr, gefolgt von einem Strichpunkt und einem Leerzeichen. Dann die Bandnummer, gefolgt von einem Doppelpunkt und eine Leerzeichen. Die Heftnummer wird bei fortlaufend nummerierten Zeitschriften nicht angegeben. Die komplette Anfangs- und Endseite werden mit einem Halbgeviertstrich (= Bindestrich, –) aber ohne Leerzeichen verbunden. Am Ende erfolgt ein Punkt und ein weicher Zeilenumbruch, um eine DOI-Nummer hinzuzufügen, bzw. ein normaler Zeilenumbruch, um mit dem nächsten Zitat fortzufahren.
□ *DOI-Nummer*: Die DOI-Nummern werden ohne Leerzeichen eingefügt gefolgt von einem normalen Zeilenumbruch. DOI-Nummern für das komplette Literaturverzeichnis können auch auf einmal auf der Internetseite http://www.crossref.org/SimpleTextQuery nach einmaliger Registrierung eingefügt und zugeordnet werden.

Die einreichenden Autoren sind verantwortlich für korrekte Daten und exakte Formatierung der Literaturzitate.

### Kriterien für eine sofortige Ablehnung

Die Schriftleitung behält sich vor, eingereichte Arbeiten auch ohne Begutachtungsverfahren abzulehnen, insbesondere bei folgenden Mängeln:

#### Sprache & Zeitschriftenstil

Arbeiten mit groben sprachlichen oder formalen Mängeln werden nicht begutachtet.

#### Ethische Stellungnahmen

Arbeiten ohne ausreichende Stellungnahme zu Interessenskonflikten, Menschenrechten (und Einverständniserklärung der Patienten) bzw. Tierrechten werden nicht bearbeitet.

#### Substanzcharakterisierung

Arbeiten ohne ausreichende analytische Untersuchung (z. B. ^13^C-NMR für neue Substanzen bzw. Chromatogramm des verwendeten Pflanzenextrakts) werden abgelehnt.

#### *In-vitro* antioxidative Wirkung

Ein Routine-Überprüfung auf in allen Pflanzen vorhandene *in-vitro*-Antioxidativität hat ohne Hinweis auf eine entsprechende *in-vivo*-Wirkung wenig Aussagekraft.

#### Diverses

Routinebestimmungen von LD_50_-Werten für neue Substanzen sind ethisch nicht vertretbar.

#### Fehlende Literaturzitate

Manuskripte ohne Diskussion und Zitierung relevanter Arbeiten werden sofort abgelehnt.

#### Spectrophotometrische und elektroanalytische Messmethoden, Schicht-Chromatographie

Wurden zu einer analytischen Problemstellung bereits moderne chromatographische Methoden publiziert, ist eine Veröffentlichung in der Scientia Pharmaceutica nur in gut begründeten Ausnahmefällen möglich.

#### Nicht offizinelle Naturstoffe

Arbeiten über die Verwendung oder Erprobung von nicht registrierten bzw. zugelassenen Naturstoffen als Hilfsstoff in neuen Formulierungen werden grundsätzlich abgelehnt.

#### Gleichzeitige quantitative Bestimmung mehrerer Substanzen

Autoren müssen den wissenschaftlichen Nutzen und die Neuigkeit in Vergleich zu bestehenden Methoden darlegen, insbesondere wenn nur eine neue Substanz zu einer bestehenden Kombination hinzugefügt wird oder wenn die Substanzen von bereits bekannten Kombinationen getauscht werden (z.B.; A+B+C wenn A+B, B+C und A+C bereist bekannt sind).

#### Plagiate und Mehrfachpublikationen

Komplette und teilweise Plagiate sowie Einreichungen bereits (größtenteils) veröffentlichter Manuskripte werden abgelehnt. Die Schriftleitung behält sich die Sperrung der involvierten Autoren für weitere Einreichungen vor.
